# Impact of COVID-19 on corneal endothelial density and morphology: a cross-sectional study in convalescent patients

**DOI:** 10.3389/fmed.2026.1878263

**Published:** 2026-08-27

**Authors:** Ling Zhang, Wenjian Shi, Xiumei Guo, Runhua Lv, Huilin Guan, Feng Zheng, Yingying Gao

**Affiliations:** 1 Department of Ophthalmology, The Second Affiliated Hospital of Fujian Medical University, Quanzhou, Fujian, China; 2 Department of Neurosurgery, The Second Affiliated Hospital of Fujian Medical University, Quanzhou, Fujian, China

**Keywords:** anterior segment parameter, corneal endothelium cell, COVID-19, SARS-CoV-2, specular microscopy

## Abstract

**Aim:**

This study aimed to investigate the effect of COVID-19 infection on corneal endothelial cell density and morphology, and to analyze the related factors.

**Methods:**

This cross-sectional study included patients who underwent corneal endothelial examination at our institute from August 2022 to May 2023. Patients were categorized into COVID-19 recovery group and non-COVID-19-infection group according to their SARS-CoV-2 infection history. Endothelial cell density (ECD), s tandard deviation of cell area (SD-Area), average cell area (ACA), and hexagonal cell percentage (HEX), among others, were compared between the two groups. To account for confounding factors, univariate analysis assessed the relationship between COVID-19 infection, demographics, medical history, and corneal endothelial cell characteristics. Subsequently, multiple linear regression pinpointed independent factors linked to cell density and morphology.

**Results:**

Three hundred seventy-eight patients recovering from COVID-19 and 382 individuals without a history of COVID-19 infection were included in the study. Analysis of corneal endothelial parameters demonstrated a statistically significant decrease in ECD (*p* < 0.001) and HEX (*p* = 0.005) in the COVID-19 recovery group compared to the non-COVID-19 infection group. Additionally, higher SD-Area (*p* < 0.001), increased ACA (*p* < 0.001), and greater MAX (*p* < 0.001) were observed in the COVID-19 recovery group. Multiple linear regression analysis suggested that COVID-19 infection history and advanced age were independent factors associated with decreased corneal endothelial density and irregular morphology.

**Conclusion:**

A history of COVID-19 infection was found to be significantly associated with decreased corneal endothelial cell density and altered morphology in convalescent patients. While these findings suggest potential long-term ocular surface implications, further prospective studies with pre-infection baselines are warranted to establish causality and to define specific, evidence-based guidelines for perioperative corneal endothelial protection in this population.

## Introduction

Severe Acute Respiratory Syndrome Coronavirus 2 (SARS-CoV-2) causes the 2019 coronavirus disease (COVID-19). Since its discovery in 2019, more than 767 million confirmed cases have been reported worldwide, with over 6.9 million deaths, making it a significant public health event in modern history (1). Infected individuals may exhibit various symptoms such as fever, cough, and myalgia, with severe cases potentially developing interstitial pneumonia or even fibrosis in both lungs (2). COVID-19 can also affect multiple organs and tissues throughout the body, including the brain, nerves, gastrointestinal tract, liver, kidneys, and eyes (3).

Studies have shown that SARS-CoV-2 can replicate and spread through contact with ocular mucous membranes exposed to viral aerosols in the air (4). Keratoconjunctivitis can be the initial symptom of COVID-19, or it can occur days to weeks after the onset of systemic symptoms (5). Regardless of the route, the cornea and conjunctiva are considered to be one of the target organs attacked by the novel coronavirus. The cornea is a critical component of the eye, with its transparency and refractive power being essential for ocular physiological functions (6). Endothelial cells, situated within the innermost layer of the cornea, establish a physical barrier delineating the interface between the Descemet membrane and the aqueous humor (7). Their function is to preserve corneal transparency by regulating the balance of ion concentration and osmotic pressure. Severe reduction or morphological changes in corneal endothelial cell density can lead to corneal edema and loss of transparency (8). Therefore, studying the impact of COVID-19 on the density and morphology of corneal endothelial cells and its mechanisms is of significant importance.

Recent studies have shown that patients during the symptomatic phase of COVID-19 have reduced corneal endothelial cell density (ECD) and hexagonal cell percentage (HEX) compared to healthy individuals (9, 10). However, these studies were limited by small sample sizes and did not include data analysis during the recovery phase. We therefore hypothesized that COVID-19 convalescent patients would exhibit persistent reductions in ECD and HEX, as well as alterations in other morphological parameters, compared to uninfected controls, and that clinical factors such as infection history and age would independently influence these changes. Consequently, this study retrospectively analyzed and compared corneal endothelial cell parameters between COVID-19 patients in the recovery phase and uninfected individuals. Additionally, it investigated influencing factors with the aim of gaining a deeper understanding of the potential impact of COVID-19 on corneal endothelial cells. This research seeks to provide theoretical support for further studies on the effects of COVID-19 on the health of the ocular surface and cornea.

## Methods

### Study design and subject

This cross-sectional study collected medical records from the Department of Ophthalmology at the Second Affiliated Hospital of Fujian Medical University between August 2022 and April 2023. The period from August 2022 to May 2023 was selected because it corresponded to a major post-omicron wave in our region, ensuring a sufficient sample of convalescent patients with documented infection during a stable phase of the pandemic. This study followed the Helsinki Declaration and received ethical approval from the hospital’s Medical Ethics Committee (No. 281, 2023). Patients were divided into COVID-19 recovery and non-COVID-19 infection groups based on SARS-CoV-2 infection history. The COVID-19 recovery group included individuals with symptomatic SARS-CoV-2 infection, confirmed by RT-PCR testing, who recovered and tested negative for at least 2 months. The non-COVID-19 infection group comprised patients with no COVID-19 infection history, no related symptoms, and negative PCR results within 1 month prior to enrollment. Exclusion criteria included a history of ocular trauma, prior ocular surgery (including intraocular injections and laser procedures), corneal diseases, glaucoma or history of intraocular pressure-lowering treatments, long-term ocular medication use, severe liver or kidney dysfunction, coagulation disorders, and inability to provide definitive COVID-19 infection history evidence. Notably, patients who had undergone intravitreal injections, peripheral iridotomy, or any form of corneal laser surgery were strictly excluded to eliminate potential iatrogenic impacts on the corneal endothelium.

### Medical record

Clinical data, including age, gender, and medical history, were retrieved through the hospital’s electronic medical record system. An epidemiological survey recorded COVID-19 infection history, positive nucleic acid test days, and the interval from symptom recovery to ophthalmic examination.

### Ocular examination

The study assessed best-corrected visual acuity (BCVA) and conducted anterior segment examinations using a slit-lamp microscope (SL-D7, Topcon) (11). Fundus photography was performed with a non-mydriatic fundus camera (CR-2 PLUS AF, Canon). Central corneal endothelial parameters were measured using non-contact specular microscopy (EM-3000, TOMEY). For each eye, three consecutive images were captured, and the average values were analyzed to ensure reproducibility. Parameters included ECD (cell/mm^2^), standard deviation of cell area (SD-Area), average cell area (ACA, μm^2^), coefficient of variation in cell area (CV, %), HEX (%), maximum cell area (MAX, μm^2^), minimum cell area (MIN, μm^2^), and central corneal thickness (CCT, μm). To maintain high data quality, all automated cell boundary identifications were manually verified by a masked investigator. Only images containing at least 100 identifiable cells were included for statistical analysis (12). An optical biometer (LENSTAR LS 900, HAAG-STREIT) assessed anterior chamber depth (ACD, mm) and white to white (WTW, mm).

### Study design and subject

Only right eye data from subjects were analyzed. SPSS Statistics 25.0 was used for data analysis, with GraphPad Prism 9 and Biorender for visualization. Continuous variables were described using mean ± SD and median (IQR). The Shapiro–Wilk test evaluated data normality, and Levene’s test assessed variance homogeneity. Student’s *t*-test was used for normally distributed variables, and the Mann–Whitney *U* test for non-normally distributed variables. Categorical data were compared using the Chi-square test. Effect sizes were calculated to quantify the magnitude of differences between groups: Cohen’s *d* was reported for parametric Student’s *t*-tests, and rank-biserial correlation r was reported for non-parametric Mann–Whitney *U* tests.

**Figure 1:**
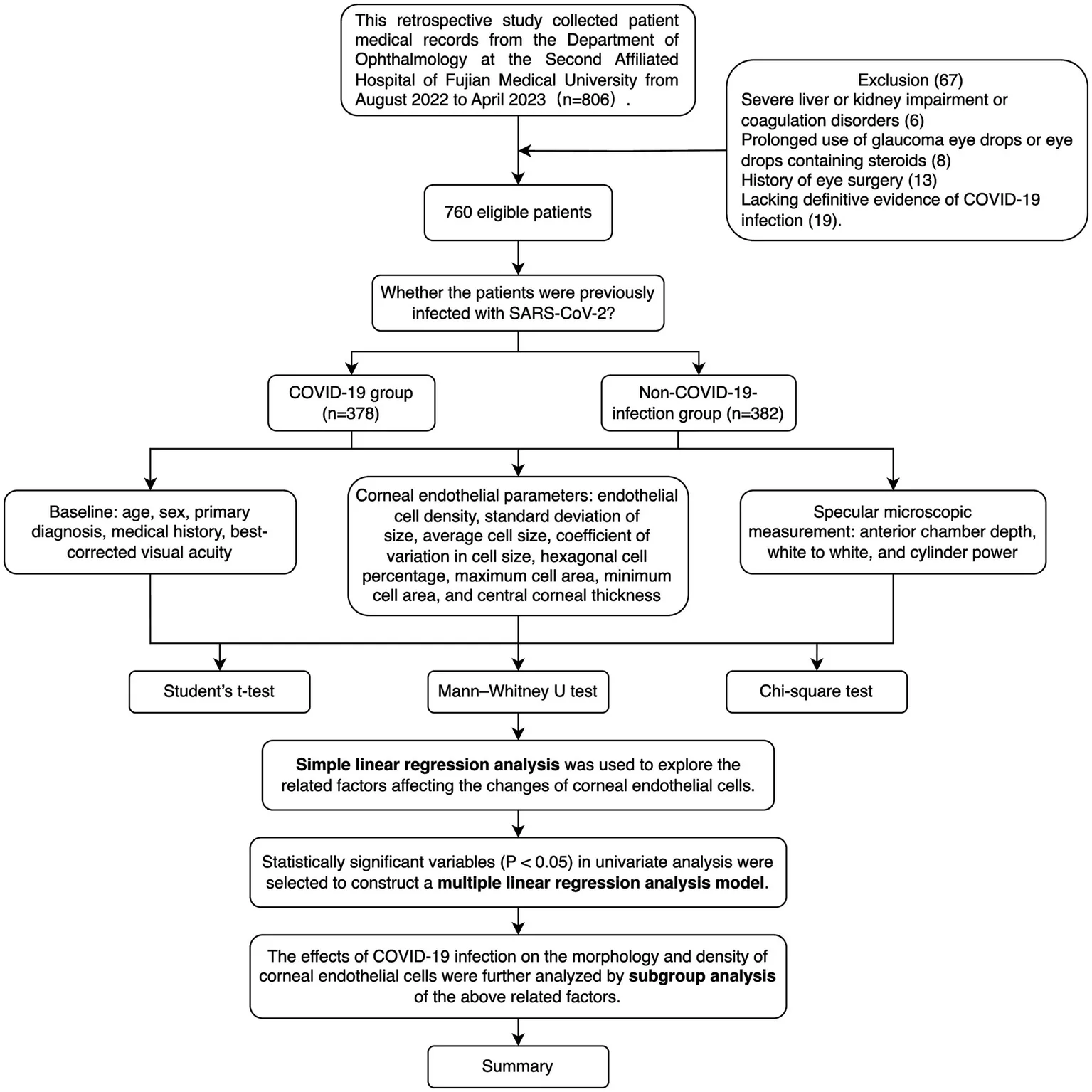
Research technology roadmap of the patient selection process and statistical analysis method.

Simple linear regression analysis was first conducted for univariate analysis of COVID-19 infection history, gender, age, major diagnosis, medical history, and BCVA. Significant variables (*p* < 0.05) were subjected to multiple linear regression analysis to identify independent correlation factors. Subgroups were established based on different age, gender, and major diagnosis to further analyze the impact of related factors on COVID-19 infection-induced changes in corneal endothelial cell morphology and density. The technical route of patient screening process and statistical analysis method in this study is shown in Figure 1. Statistical significance was defined as *p* < 0.05.

## Results

This study included a total of 760 patients, among which 378 (49.7%) were COVID-19 recovery patients, and the remaining 382 (50.3%) comprised the non-COVID-19-infection group. The average age of the patients was 64.8 (± 12.5) years, with 317 male patients (41.7%). The demographic characteristics of the patients at the time of their visit and general ophthalmic examination features were detailed in Table 1.

**Table 1:** Demographic and clinical characteristics between COVID-19 recovery group and non-COVID-19 infection group.

Variables	Total (*n* = 760)	COVID-19 recovery group (*n* = 378)	Non-COVID-19 infection group (*n* = 382)	*p-*value
Baseline				
Age, years, mean ± SD	64.8 ± 12.5	64.0 ± 11.0	65.6 ± 13.7	**0.032**
Sex, man, *n* (%)	317 (41.7%)	151 (39.9%)	166 (43.5%)	0.339
Disorder of lens, *n* (%)	623 (82.0%)	315 (83.3%)	308 (80.6%)	0.346
Disorder of retina, *n* (%)	132 (17.4%)	63 (16.7%)	69 (18.1%)	0.633
Diabetes mellitus, *n* (%)	88 (11.6%)	43 (11.4%)	45 (11.8%)	0.910
Hypertension, *n* (%)	163 (21.4%)	84 (22.2%)	79 (20.7%)	0.659
DM and HTN combined, *n* (%)	114 (15.0%)	51 (13.5%)	63 (16.5%)	0.265
Clinical characteristics				
BCVA, mean ± SD	0.4 ± 0.3	0.4 ± 0.2	0.4 ± 0.3	0.390
Duration of positive SARS-CoV-2 PCR test, days, mean ± SD	—	9.4 ± 3.1	—	—
Time elapsed since the PCR test was negative, days, mean ± SD	—	63.8 ± 26.4	—	—

Values in bold indicate results with statistically significant differences (*p* < 0.05). HTN, hypertension; DM, diabetes mellitus; BCVA, best-corrected visual acuity; PCR, Polymerase Chain Reaction.

The corneal ECD (2559.8 [262.5] vs. 2736.2 [238.7], *p* < 0.001) and HEX (49.0 [43.0–55.0] vs. 51.0 [46.0–55.0], *p* = 0.005) was significantly decreased in the COVID-19 recovery group compared to the non-COVID-19-infection group. The COVID-19 recovery group exhibited a significantly increased SD-Area (154.5 [134.0–179.3] vs. 142.0 [125.8–162.0], *p* < 0.001), ACA (390.0 [364.0–421.3] vs. 364.0 [345.0–387.0], *p* < 0.001) and MAX (1002.5 [870.5, 1171.0] vs. 923.5 [812.5, 1074.3], *p* < 0.001). After COVID-19 recovery, no significant difference was detected in CV (*p* = 0.134), MIN (*p* = 0.087), CCT (*p* = 0.192), ACD (*p* = 0.263), and WTW (*p* = 0.628) between the two groups (Table 2).

**Table 2:** Analysis of corneal endothelial parameters and anterior segment measurement between COVID-19 recovery group and non-COVID-19 infection group.

Variables	Total (*n* = 760)	COVID-19 recovery group (*n* = 378)	Non-COVID-19 infection group (*n* = 382)	*Z* value	*p-*value	Effect size
ECD^*^, cell/mm^2^	2648.49 ± 265.7	2559.8 ± 262.5	2736.2 ± 238.7	−9.355	**<0.001**	0.339
SD-Area^*^	152.9 ± 31.9	159.9 ± 33.8	146.0 ± 28.2	−5.818	**<0.001**	0.211
ACA^*^, μm^2^	381.5 ± 40.5	394.8 ± 42.4	368.4 ± 33.9	−9.351	**<0.001**	0.339
CV, %	40.1 ± 6.6	40.4 ± 6.7	39.7 ± 6.4	−1.498	0.134	0.054
MAX^*^, μm^2^	1016.9 ± 255.9	1049.6 ± 261.7	984.6 ± 246.0	−4.372	**<0.001**	0.159
MIN, μm^2^	118.9 ± 21.3	120.6 ± 22.3	117.2 ± 20.2	−1.714	0.087	0.062
HEX^*^, %	49.8 ± 7.8	48.9 ± 7.9	50.6 ± 7.5	−2.784	**0.005**	0.101
CCT, μm	528.9 ± 35.0	530.3 ± 33.6	527.5 ± 36.3	−1.305	0.192	0.047
ACD, mm	2.6 ± 0.5	2.6 ± 0.3	2.5 ± 0.5	−1.119	0.263	0.041
WTW, mm	11.5 ± 0.6	11.5 ± 0.6	11.5 ± 0.7	−0.395	0.268	0.013

Values reported as Mean ± SD. Values in bold indicate results with statistically significant differences (*p* < 0.05). *Statistically significant difference when *p* < 0.05 compared with the non-COVID-19 infection group. ECD, endothelial cell density; SD-Area, standard deviation of cell area; ACA, average cell area; CV, coefficient of variation in cell area; MAX, maximum cell area; MIN, minimum cell area; HEX, hexagonal cell percentage; CCT, central corneal thickness; ACD, anterior chamber depth; WTW, white to white.

Due to the impact of various factors on the density and morphology of corneal endothelial cells, a simple linear regression analysis and univariate analysis were conducted to investigate the relationship between these confounding variables and their changes. In univariate analysis, COVID-19 infection history (*p* < 0.001), advanced age (*p* < 0.001), female (*p* = 0.001), lens related disease (*p* < 0.001) and history of diabetes (*p* = 0.021) were significantly associated with corneal ECD decreased. Multivariate analysis showed that history of COVID-19 infection (*p* < 0.001), advanced age (*p* < 0.001), and female (*p* = 0.012) were significantly associated with corneal ECD decreased (Table 3). Univariate analysis showed that corneal endothelial HEX was significantly associated with history of COVID-19 infection (*p* = 0.003), advanced age (*p* = 0.009), lens related disease (*p* < 0.001), and history of combined hypertension and diabetes (*p* < 0.001). Multivariate analysis showed that a history of COVID-19 infection (p = 0.003), lens-related disease (*p* = 0.006), combined history of hypertension and diabetes (*p* < 0.001) were significantly associated with corneal endothelial HEX decreased (Table 4).

**Table 3:** Univariate and multivariate analysis of influencing factors on corneal endothelial cell density (ECD).

Variables	Univariate analysis			Multivariate analysis		
	Standardization coefficient	95% CI	*p*-value	Standardized coefficient	95% CI	*p*-value
History of COVID-19 infection^**^	−0.33	−212.12, −140.67	**<0.001**	−0.34	−216.75, −146.74	**<0.001**
Age^**^	−0.12	−5.47, −2.48	**<0.001**	−0.19	−5.69, −2.50	**<0.001**
Gender, Female^**^	−0.10	−105.03, −28.82	**0.001**	−0.09	−81.44, −10.00	**0.012**
Major diagnosis, lens related disease^*^	−0.12	−138.30, −40.61	**<0.001**	−0.05	−80.96, 17.01	0.200
Diabetes mellitus^*^	−0.08	−67.78, 51.16	**0.021**	−0.03	−84.75, 29.72	0.346
Hypertension	−0.03	−70.41, 21.18	0.784	—	—	—
DM and HTN combined	−0.02	−54.95, 51.11	0.292	—	—	—
BCVA	0.03	−32.65, 91.17	0.354	—	—	—

This table employs simple linear regression analysis and multiple linear regression analysis to explore the factors influencing corneal endothelial cell density. Values in bold indicate results with statistically significant differences (*p* < 0.05). An asterisk (*) denotes significance at *p* < 0.05 in univariate analysis only, while a double asterisk (**) indicates significance at *p* < 0.05 in both univariate and multivariate linear regression analyses. CI, confidence interval; HTN, hypertension; DM, diabetes mellitus; BCVA, best-corrected visual acuity.

**Table 4:** Univariate and multivariate analysis of the effects of influencing factors on corneal endothelial hexagonal cell percentage (HEX).

Variables	Univariate analysis			Multivariate analysis		
	Standardization coefficient	95% CI	*p*-value	Standardized coefficient	95% CI	*p*-value
History of COVID-19 infection^**^	−0.11	−2.79, −0.60	**0.003**	−0.11	−2.76, −0.58	**0.003**
Age^**^	−0.09	−0.10, −0.01	**0.009**	−0.08	−0.10, 0.001	**0.045**
Gender, Female	−0.02	−1.45, 0.79	0.561	—	—	—
Major diagnosis, lens related disease^**^	0.73	−4.25, −1.40	**<0.001**	−0.11	−3.67, −0.64	**0.006**
Diabetes mellitus	−0.06	−3.24, 0.23	0.089	—	—	—
Hypertension	−0.03	−1.87, 0.81	0.435	—	—	—
DM and HTN combined^**^	0.14	1.52, 4.58	**<0.001**	0.14	1.47, 4.71	**<0.001**
BCVA	0.06	−0.36, 3.26	0.115	—	—	—

This table employs simple linear regression analysis and multiple linear regression analysis to explore the factors influencing corneal endothelial hexagonal cell percentage. Values in bold indicate results with statistically significant differences (*p* < 0.05). An asterisk (*) denotes significance at *p* < 0.05 in univariate analysis only, while a double asterisk (**) indicates significance at *p* < 0.05 in both univariate and multivariate linear regression analyses. CI, confidence interval; HTN, hypertension; DM, diabetes mellitus; BCVA, best-corrected visual acuit.

Subgroup analysis was further conducted based on gender, age, primary diagnosis, medical history, and BCVA. In these subgroups categorized by gender (male, female), age (below 60, 61–79), primary diagnosis (lens disorder, retinal disorders), medical history [diabetes mellitus (DM), hypertension (HTN), DM and HTN combined], and BCVA (less than 10/20, greater than 10/20), ECD (*p* < 0.05) and HEX (*p* < 0.05) were significantly reduced (Table 5), while ACA (*p* < 0.05) and MAX (*p* < 0.05) were significantly increased in the COVID-19 recovery group compared to the non-COVID-19-infection group. In the COVID-19 recovery group, increased CV was detected in individuals under 60 years old (*p* = 0.01) and in males (*p* = 0.05), with a significant increase in MIN in individuals with BCVA greater than 10/20 (*p* = 0.002) and in those under 60 years old (*p* = 0.02) compared to non-COVID-19-infection group (Supplementary Table 1).

**Table 5:** Subgroup analyses of corneal endothelial cell density (ECD) and hexagonal cell percentage (HEX) between COVID-19 recovery group and non-COVID-19 infection group.

Subgroups	Group	ECD	*p*-value	HEX	*p*-value
Age	≤ 60 & COVID-19 (*n* = 139)	2599.3 ± 273.6	**<0.001**	49.4 ± 9.0	**0.028**
	& Non-CoV-19 (*n* = 122)	2802.1 ± 210.9		52.3 ± 7.5	
	61–79 & COVID-19 (*n* = 221)	2538.4 ± 248.1	**<0.001**	48.7 ± 7.0	**0.002**
	& Non-CoV-19 (*n* = 191)	2742.8 ± 222.6		50.8 ± 6.6	
	≥ 80 & COVID-19 (*n* = 18)	2509.9 ± 324.2	0.229	47.7 ± 10.5	0.958
	& Non-CoV-19 (*n* = 69)	2601.4 ± 274.4		48.8 ± 9.4	
Gender	Male & COVID-19 (*n* = 151)	2582.6 ± 291.0	**<0.001**	48.9 ± 8.4	**0.035**
	& Non-CoV-19 (*n* = 166)	2782.9 ± 255.4		50.9 ± 7.9	
	Female & COVID-19 (*n* = 227)	2544.7 ± 241.1	**<0.001**	48.9 ± 7.6	0.071
	& Non-CoV-19 (*n* = 216)	2700.3 ± 219.0		50.4 ± 7.2	
BCVA	≤10/20 & COVID-19 (*n* = 221)	2572.6 ± 257.3	**<0.001**	48.8 ± 7.8	**0.040**
	& Non-CoV-19 (*n* = 219)	2714.4 ± 248.9		50.5 ± 7.3	
	>10/20 & COVID-19 (*n* = 163)	2542.9 ± 269.1	**<0.001**	49.1 ± 8.2	0.067
	& Non-CoV-19 (*n* = 157)	2764.5 ± 218.7		50.7 ± 7.7	
Primary diagnosis	Lens & COVID-19 (*n* = 315)	2550.7 ± 260.9	**<0.001**	48.4 ± 7.9	**0.009**
	& Non-CoV-19 (*n* = 308)	2716.8 ± 239.4		50.2 ± 7.5	
	Retinal & COVID-19 (*n* = 63)	2605.8 ± 267.7	**<0.001**	51.3 ± 7.6	0.383
	& Non-CoV-19 (*n* = 69)	2809.9 ± 222.6		52.8 ± 7.1	
Medical history	HPN & COVID-19 (*n* = 84)	2559.8 ± 226.2	**0.001**	48.8 ± 7.8	0.613
	& Non-CoV-19 (*n* = 82)	2686.2 ± 253.7		49.6 ± 7.1	
	DM & COVID-19 (*n* = 44)	2519.9 ± 323.0	**<0.001**	49.0 ± 8.1	0.464
	& Non-CoV-19 (*n* = 43)	2765.2 ± 264.5		47.8 ± 6.6	
	HPN&DM & COVID-19 (*n* = 52)	2546.1 ± 318.5	**<0.001**	51.2 ± 8.0	0.166
	& Non-CoV-19 (*n* = 62)	2737.1 ± 214.9		53.2 ± 6.9	

Values reported as mean ± SD. Values in bold indicate results with statistically significant differences (*p* < 0.05). *Statistically significant difference when *p* < 0.05 compared with the non-COVID-19 infection group. ECD, endothelial cell density; HEX, hexagonal cell percentage; HTN, hypertension; DM, diabetes mellitus.

**Figure 2:**
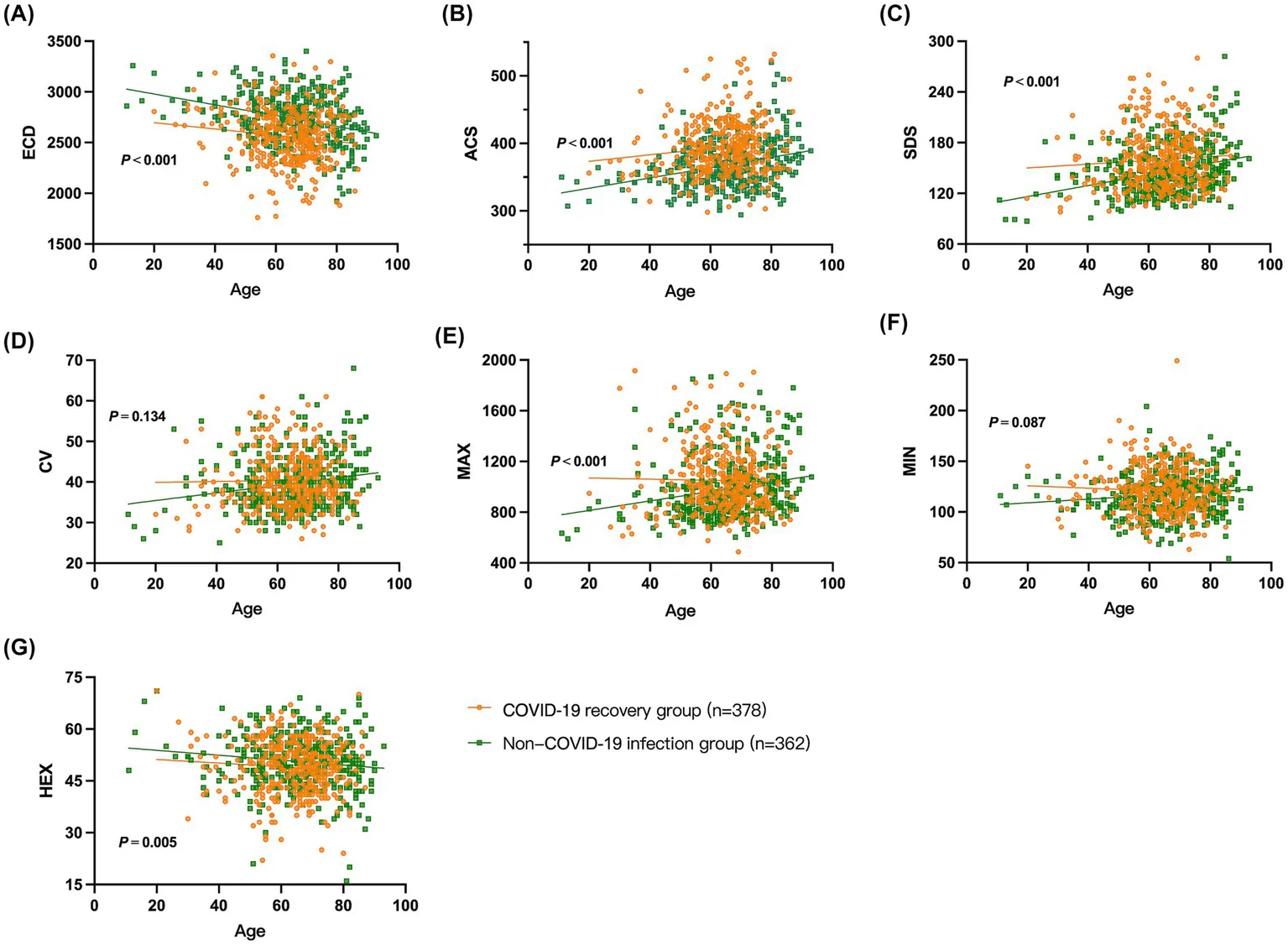
Scatterplots for the correlations between ECD **(A)**, SD-Area **(B)**, ACA **(C)**, CV **(D)**, MAX **(E)**, MIN **(F)**, HEX **(G)** and age in COVID-19 recovery group and non-COVID-19-infection group. ECD, endothelial cell density; ACA, average cell area; SD-Area, standard deviation of cell area; CV, coefficient of variation in cell area; MAX, maximum cell area; MIN, minimum cell area; HEX, hexagonal cell percentage.

As mentioned previously, a history of COVID-19 infection and advanced age are independent factors affecting the density and morphological changes of corneal endothelial cells. Therefore, with age as the independent variable, scatter plots of various parameters of corneal endothelial cells were plotted, as shown in Figure 2. With increasing age, there is a decreasing trend in ECD, MAX, MIN, and HEX in the COVID-19 recovery group, while ACA, SD-Area, and CV show an increasing trend. In the group uninfected with COVID-19, ECD and HEX exhibit a decreasing trend with age, whereas ACA, SD-Area, CV, MAX, and MIN demonstrate an increasing trend.

## Discussion

This cross-sectional study evaluated the association between a history of COVID-19 infection and corneal endothelial parameters, as well as the potential factors linked to these alterations. Compared with individuals without a history of infection, convalescent COVID-19 patients exhibited significantly lower corneal endothelial cell density (ECD) and hexagonal cell percentage (HEX), along with higher standard deviation of cell area (SD-Area), average cell area (ACA), and maximum cell area (MAX). Multivariable analysis further indicated that a history of COVID-19 infection, advanced age, female sex, lens-related diseases, diabetes mellitus, and poorer visual acuity were associated with these endothelial changes. Collectively, these observations point to a persistent relationship between past COVID-19 infection and alterations in corneal endothelial morphology, highlighting the need to better understand ocular surface health during post-viral recovery.

**Figure 3:**
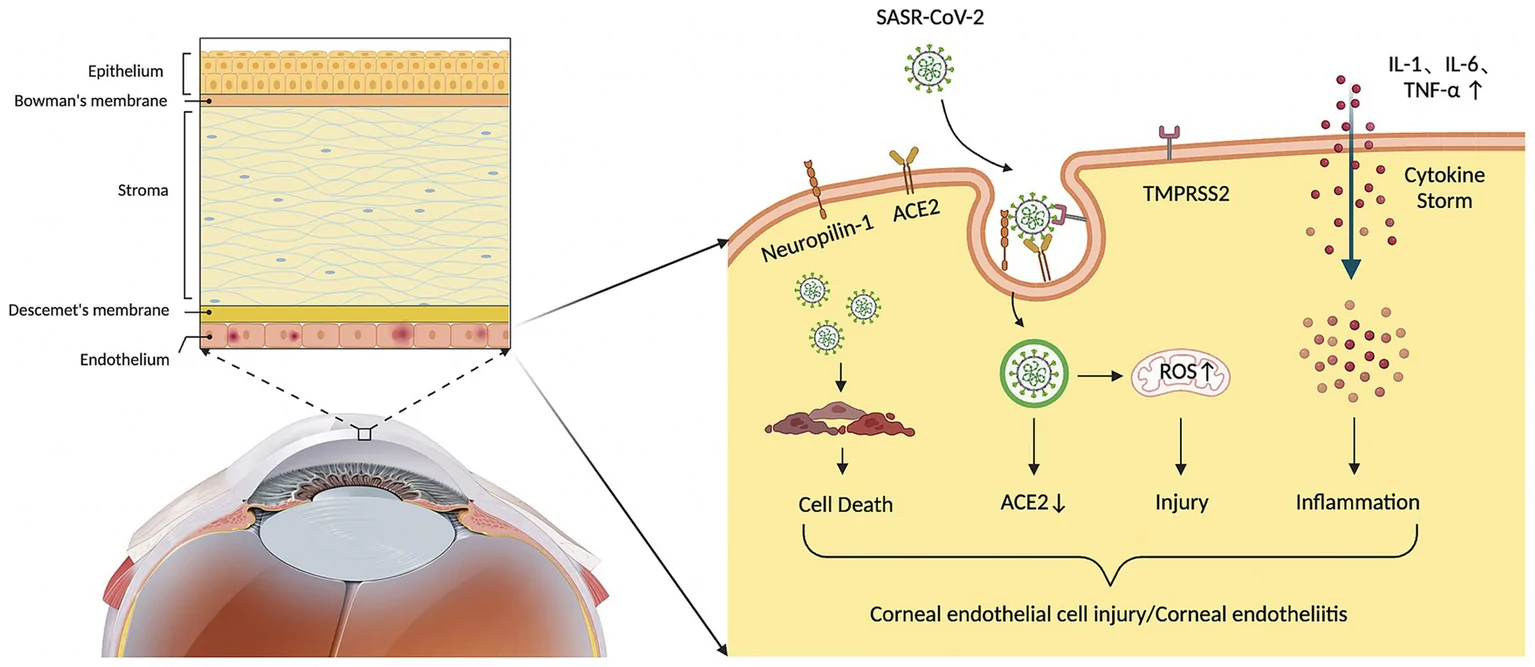
Possible mechanisms of the effect of SARS-CoV-2 on corneal endothelial cells. Created in BioRender. [Yingying Gao] (2026). https://app.biorender.com/illustrations/623dd857655e20ca9d641da6?slideId=b8969d15-bc1a-415f-a9b1-10a8bdf2ada4. The corneal endothelium, situated in the deepest layer of the cornea and in direct contact with the aqueous humor, may be susceptible to direct damage from SARS-CoV-2 present in the aqueous humor, leading to cellular apoptosis. The entry of SARS-CoV-2 into host cells is facilitated by the binding of its surface protein receptor-binding domain to the ACE2 receptor on the cell surface, with assistance from TMPRSS2 and Neuropilin-1. Following attachment to ACE2, a decrease in ACE2 expression occurs alongside an increase in Ang II levels, resulting in significant oxidative stress reactions and eventual cellular damage. Moreover, infection with SARS-CoV-2 has the potential to induce a cytokine storm, resulting in elevated levels of pro-inflammatory cytokines such as IL-6, IL-1, and tumor necrosis factor-alpha. This cascade of events may lead to inflammation in corneal endothelial cells, ultimately causing damage or corneal endotheliitis (ACE2, angiotensin-converting enzyme 2; TMPRSS2, transmembrane protease serine 2; IL-6, interleukin-6; ROS, reactive oxygen species).

Corneal endothelial cells maintain corneal transparency through barrier and active pump functions (13). While SARS-CoV-2 entry factors (such as ACE2 and TMPRSS2) are expressed on ocular tissues, the exact mechanism of virus-induced endothelial damage remains under investigation (14). roposed mechanisms primarily involve a combination of direct viral cytopathic effects and indirect immune-mediated ocular surface inflammation (Figure 3) (15–17). Transient local endotheliitis or systemic cytokine cascades during acute infection may trigger endothelial cell apoptosis, leading to secondary cell enlargement and loss of hexagonality during the repair phase (18–20).

The chronological window of data collection in this study, spanning from August 2022 to April 2023, directly coincided with the peak of the SARS-CoV-2 Omicron variant wave in China. Accumulating evidence indicates that the Omicron variant possesses a distinct immunological and inflammatory profile compared to previous ancestral strains or the Delta variant, characterized generally by lower systemic virulence but a higher propensity for upper respiratory colonization. Although the general systemic inflammatory response induced by Omicron might be less severe, its unprecedentedly massive infection scale during this period underscores its potential, yet unrecognized, impact on ocular microenvironments.

Within this specific epidemiological context, the potential confounding roles of acute-phase ocular manifestations and therapies must be acknowledged. Unlike superficial conjunctivitis, clinical subentities like transient anterior uveitis can expose the corneal endothelium to direct inflammatory insults via the aqueous humor. Similarly, acute-phase systemic or topical corticosteroid treatments can independently influence endothelial morphology. Because most patients initially self-isolated or were managed in non-ophthalmic wards, precise registries tracking these acute ocular signs and steroid dosages were retrospecitively unavailable. To minimize such iatrogenic confounding, our study rigorously excluded individuals using prolonged glaucoma or steroid-containing eye drops. These persistent alterations still suggest a significant association between COVID-19 history and endothelial impairment, though partitioning the exact impacts of direct viral cytopathic effects, secondary uveitis, and acute therapies requires future prospective validation.

Endothelial cell density (ECD) represents a pivotal metric for assessing the functional capacity and condition of the corneal endothelium (12). Our study showed a notable decrease in ECD among individuals convalescing from COVID-19, may be due to endothelial dysfunction and cell apoptosis related to the viral immune response and inflammation (9). This has implications for preoperative ECD assessment in post-COVID-19 patients before internal eye surgeries (21, 22). Similar to the results of this study, previous studies have found that, compared with healthy controls, decreased ECD can be observed during the disease active phase of COVID-19 infection (23, 24). However, this study was conducted post-symptomatically, with all participants having recovered from COVID-19 for over 2 months, suggesting that the detrimental effects of the infection on corneal endothelial cells are enduring and potentially irreversible.

Hexagonal cell percentage (HEX), a measure of morphological variation, also decreased in post-COVID-19 patients, indicating compromised cell stability or migration. Loss of hexagonal cell morphology can impair the corneal endothelial barrier function, potentially leading to corneal decompensation (25, 26). Recent studies have detected a decrease in HEX when compared to healthy controls, not only during the symptomatic phase of COVID-19 but also 1 month post-recovery, aligning with the findings of this investigation. However, the studies were limited by a small sample size and a brief observation period following convalescence (9, 27).

The study found no significant difference in coefficient of variation (CV) between the COVID-19 recovery and uninfected groups. Previous studies indicate that COVID-19 patients have significantly higher corneal endothelial cell variability compared to healthy individuals, especially in the early stages of the disease, with a pronounced increase observed in severe cases over the course of a month (9, 10). The two studies’ divergent results from ours may be due to corneal endothelial cells initiating reparative processes post-injury, with adjacent cells migrating to restore the monolayer structure, potentially normalizing cell distribution in COVID-19 convalescents after 2 months (10, 27).

Advanced age, female gender, lens-related diseases, diabetes mellitus, and poor vision were associated with alterations in corneal endothelial cell density and morphology. Age-related declines in ECD and HEX, and increases in ACA and CV, are well-documented. Specifically, the corneal ECD declines by an average of 0.3–0.5% annually, from approximately 5,000 cells/mm^2^ at birth to about 2,500 cells/mm^2^ in old (23, 24, 28). Gender differences in corneal endothelial cells may be influenced by hormonal changes, particularly in middle-aged and older women (21). Diabetes can affect the morphological structure of the corneal endothelium, potentially damaging the hexagonal structure and impacting cell repair and compensation (29, 30). The study’s findings indicate that lens-related diseases may impact corneal endothelial cells, with age-related cataracts potentially influencing corneal endothelial cell metabolism and function (31). Poor vision was independently associated with increased SD-Area, CV, and MAX, highlighting the importance of corneal endothelial morphology and uniform cell area for maintaining visual acuity (22).

Subgroup analyses revealed that age significantly differed between the COVID-19 recovery and uninfected groups, except for those over 80. Although our findings showed an association between poorer vision and altered endothelial parameters (higher SD-Area, CV, and MAX), this correlation should be interpreted with caution. In the absence of clinical or subclinical corneal edema, endothelial changes alone are unlikely to directly impair visual acuity. Instead, the observed reduction in vision is likely attributable to age-related factors, such as cataract progression or other ocular comorbidities, rather than a direct causative impact from the corneal endothelium (32). Further analysis indicated that in COVID-19 recoverers, males and those under 60 had a higher CV, suggesting gender and age-related physiological and immune responses to the virus may affect corneal endothelial variability.

Subgroup analysis of corneal endothelial cells’ coefficient of variation (CV) in COVID-19 recovery patients revealed significant increases in males and those under 60, possibly due to gender and age-related physiological responses to the virus, which may result in greater variability in corneal endothelial cells among these demographics (33, 34). This suggests that the immune response to COVID-19, which can vary by age and gender, might have direct or indirect effects on corneal endothelial cells, contributing to the observed increase in CV.

### Limitation

Several limitations of this study should be acknowledged. First, the retrospective design without pre-COVID-19 baseline measurements precludes any causal inference. Second, due to data tracking constraints during the acute infection phase, individual clinical records regarding acute ocular manifestations (e.g., anterior uveitis) and corticosteroid therapies were unavailable. Third, despite stringent control group criteria (negative RT-PCR within 1 month and no symptomatic history), we cannot definitively exclude asymptomatic SARS-CoV-2 infection, particularly given the high transmissibility of Omicron variants during the study period. This potential misclassification may have attenuated the observed differences. Fourth, the clinical severity of COVID-19 (such as hospitalization or the need for oxygen therapy) was not systematically recorded in our electronic registry. Given that vaccination and viral lineages significantly modulate systemic and localized inflammatory responses, the absence of these variables constitutes a potential confounder. Consequently, these findings should be interpreted with caution, and future prospective, multi-center studies with comprehensive baseline data are warranted.

## Conclusion

In conclusion, a history of COVID-19 infection was significantly associated with reduced corneal endothelial cell density and altered morphology in convalescent patients. Given the observational and cross-sectional nature of this study, these findings reflect a clinical association rather than a direct causal relationship. While our results suggest that clinicians should remain cognizant of potential long-term alterations in corneal health among post-COVID-19 patients, further prospective investigations with pre-infection baseline data are warranted to fully elucidate the true causality and establish precise, evidence-based guidelines for perioperative corneal endothelial protection during intraocular surgery.

## Data Availability

The raw data supporting the conclusions of this article will be made available by the corresponding author via email upon reasonable request, without undue reservation.

## Glossary

COVID-19: corona virus disease 2019
SARS-Cov-2: severe acute respiratory syndrome coronavirus 2
RBD: receptor-binding domain
ACE2: angiotensin-converting enzyme 2
TMPRSS2: transmembrane protease serine 2
BCVA: best-corrected visual acuity
RT-PCR: real-time reverse transcriptase-polymerase chain reaction
ECD: endothelial cell density
SD-Area: standard deviation of cell area
ACA: average cell area
HEX: hexagonal cell percentage
CV: coefficient of variation in cell area
MAX: maximum cell area
MIN: minimum cell area
CCT: central corneal thickness
ACD: anterior chamber depth
WTW: white to white
SD: standard deviation
DM: diabetes mellitus
HTN: hypertension
