# Supplementary material for: Impact of COVID-19 on corneal endothelial density and morphology: a cross-sectional study in convalescent patients
Source: Front Med (Lausanne). 2026 Aug 27;13:1878263. doi: 10.3389/fmed.2026.1878263 (PMC13558423; doi:10.3389/fmed.2026.1878263)
Supplement: Supplementary file 1 [file Table_1.docx]

|  | Group | ACS | *P*-value | SDS | *P*-value | CV | *P*-value |
| --- | --- | --- | --- | --- | --- | --- | --- |
| Age | ≤ 60 & COVID-19 (n=139) | 389.5±44.9 | **<0.001** | 160.2±38.3 | **<0.001** | 40.9±7.7 | **0.038** |
|  | & Non-CoV-19 (n=122) | 358.9±27.5 |  | 138.5±24.6 |  | 38.7±6.4 |  |
|  | 61-79 & COVID-19 (n=221) | 397.3±39.0 | **<0.001** | 159.5±30.5 | **<0.001** | 40.1±6.1 | 0.205 |
|  | & Non-CoV-19 (n=191) | 367.0±30.7 |  | 144.7±25.2 |  | 39.6±5.9 |  |
|  | ≥ 80 & COVID-19 (n=18) | 405.6±58.9 | 0.668 | 163.1±36.7 | 0.457 | 40.1±6.3 | 0.993 |
|  | & Non-CoV-19 (n=69) | 388.9±43.0 |  | 163.1±34.8 |  | 41.9±7.4 |  |
| Gender | Male & COVID-19 (n=151) | 392.4±47.2 | **<0.001** | 159.5±35.9 | **<0.001** | 40.3±6.9 | **0.050** |
|  | & Non-CoV-19 (n=166) | 362.6±35.6 |  | 141.1±26.9 |  | 38.9±6.2 |  |
|  | Female & COVID-19 (n=227) | 396.3±39.0 | **<0.001** | 160.2±32.3 | **0.001** | 40.5±6.6 | 0.818 |
|  | & Non-CoV-19 (n=216) | 372.8±31.8 |  | 149.8±28.7 |  | 40.3±6.5 |  |
| BCVA | ≤10/20 & COVID-19 (n=221) | 392.3±39.8 | **<0.001** | 162.0±32.9 | **<0.001** | 41.1±6.6 | 0.169 |
|  | & Non-CoV-19 (n=219) | 371.7±35.9 |  | 149.7±30.6 |  | 40.4±7.0 |  |
|  | >10/20 & COVID-19 (n=163) | 398.1±45.5 | **<0.001** | 157.2±34.8 | **<0.001** | 39.5±6.8 | 0.818 |
|  | & Non-CoV-19 (n=157) | 363.9±30.0 |  | 141.1±23.3 |  | 38.9±5.3 |  |
| Primary diagnosis | Lens & COVID-19 (n=315) | 396.1±42.3 | **<0.001** | 160.6±33.5 | **<0.001** | 40.6±6.8 | 0.405 |
|  | & Non-CoV-19 (n=308) | 371.1±34.5 |  | 148.5±28.2 |  | 40.1±6.5 |  |
|  | Retinal & COVID-19 (n=63) | 388.3±42.7 | **<0.001** | 156.6±35.3 | **<0.001** | 39.7±6.4 | 0.125 |
|  | & Non-CoV-19 (n=69) | 357.9±28.9 |  | 136.4±26.6 |  | 38.1±6.3 |  |
| Medical history | HPN & COVID-19 (n=84) | 392.6±33.2 | **0.001** | 159.2±28.3 | **0.010** | 40.6±6.0 | 0.146 |
|  | & Non-CoV-19 (n=82) | 375.7±36.4 |  | 149.7±32.4 |  | 39.7±6.5 |  |
|  | DM & COVID-19 (n=44) | 404.2±57.1 | **<0.001** | 162.5±38.4 | **0.010** | 40.4±7.0 | 0.929 |
|  | & Non-CoV-19 (n=43) | 364.8±34.7 |  | 144.4±24.1 |  | 40.2±5.5 |  |
|  | HPN&DM & COVID-19 (n=52) | 399.0±51.6 | **<0.001** | 161.7±39.9 | **0.095** | 39.7±6.5 | 0.909 |
|  | & Non-CoV-19 (n=62) | 367.6±29.9 |  | 148.4±34.1 |  | 40.3±7.8 |  |

**Supplementary table 1** Subgroup analyses of corneal endothelial average cell size (ACS), standard deviation of size (SDS), coefficient of variation in cell size (CV), maximum cell area (MAX) and minimum cell area (MIN) between COVID-19 recovery group and non-COVID-19 infection group

|  | Group | MAX | *P*-value | MIN | *P*-value |
| --- | --- | --- | --- | --- | --- |
| Age | ≤ 60 & COVID-19 (n=139) | 1067.9±275.2 | **<0.001** | 120.8±23.0 | **0.021** |
|  | & Non-CoV-19 (n=122) | 932.5±258.3 |  | 114.5±19.7 |  |
|  | 61-79 & COVID-19 (n=221) | 1039.7±254.4 | **0.006** | 120.6±22.6 | 0.382 |
|  | & Non-CoV-19 (n=191) | 986.8±231.5 |  | 117.4±19.4 |  |
|  | ≥ 80 & COVID-19 (n=18) | 1028.0±255.2 | 0.381 | 120.3±14.4 | 0.414 |
|  | & Non-CoV-19 (n=69) | 1070.5±241.4 |  | 121.7±22.5 |  |
| Gender | Male & COVID-19 (n=151) | 1051.9±254.7 | **<0.001** | 121.0±22.0 | 0.172 |
|  | & Non-CoV-19 (n=166) | 946.2±234.9 |  | 116.6±18.4 |  |
|  | Female & COVID-19 (n=227) | 1048.1±266.8 | **0.073** | 120.4±22.6 | 0.276 |
|  | & Non-CoV-19 (n=216) | 1014.1±250.8 |  | 117.7±21.5 |  |
| BCVA | ≤10/20 & COVID-19 (n=221) | 1063.7±268.3 | **0.007** | 119.6±22.6 | 0.990 |
|  | & Non-CoV-19 (n=219) | 1013.3±261.9 |  | 118.8±20.8 |  |
|  | >10/20 & COVID-19 (n=163) | 1030.9±252.3 | **0.001** | 122.0±22.0 | **0.002** |
|  | & Non-CoV-19 (n=157) | 944.7±216.4 |  | 114.9±19.1 |  |
| Primary diagnosis | Lens & COVID-19 (n=315) | 1044.4±253.5 | **0.003** | 121.3±22.5 | 0.062 |
|  | & Non-CoV-19 (n=308) | 996.7±237.6 |  | 118.0±20.7 |  |
|  | Retinal & COVID-19 (n=63) | 1075.6±300.2 | **<0.001** | 117.3±21.2 | 0.480 |
|  | & Non-CoV-19 (n=69) | 944.4±281.4 |  | 113.7±18.1 |  |
| Medical history | HPN & COVID-19 (n=84) | 1060.9±249.6 | 0.080 | 120.65±20.0 | 0.968 |
|  | & Non-CoV-19 (n=82) | 1014.3±263.2 |  | 120.4±19.5 |  |
|  | DM & COVID-19 (n=44) | 1063.4±295.7 | 0.054 | 121.3±22.3 | 0.150 |
|  | & Non-CoV-19 (n=43) | 960.4±219.5 |  | 114.7±25.0 |  |
|  | HPN&DM & COVID-19 (n=52) | 1073.7±292.7 | 0.109 | 117.9±22.5 | 0.198 |
|  | & Non-CoV-19 (n=62) | 999.6±257.1 |  | 118.1±19.9 |  |

Abbreviations: ACS, average cell size; SDS, standard deviation of size; CV, oefficient of variation in cell size; MAX, maximum cell area ; MIN, minimum cell area; HTN, hypertension; DM, diabetes mellitus. Values reported as Mean±SD. * Statistical significant difference when P < 0.05 compared to COVID-19 recovery group.
